# Morphological analysis of the skeletal development in lateral cephalometric radiographs of HIV infected children ongoing Highly Active Antiretroviral Therapy

**DOI:** 10.4317/medoral.22610

**Published:** 2018-11-21

**Authors:** Letícia-Pereira Possagno, Ademir Franco, Luiz-Renato Paranhos, Liliane-Janete Grando, Antonio-Adilson-Soares de Lima, Ilana-Sanamaika-Queiroga Bezerra, Ângela Fernandes

**Affiliations:** 1 Department of Stomatology, Federal University of Paraná, Brazil; 2 Department of Therapeutic Stomatology, Institute of Dentistry, Sechenov University, Russia; 3 Department of Preventive and Community Dentistry, School of Dentistry, Federal University of Uberlândia, Brazil; 4 Department of Pathology, Center of Health Sciences, Federal University of Santa Catarina, Brazil; 5 Department of Radiology and Imaging, Technical School of Oral Health, Federal University of Campina Grande, Brazil

## Abstract

**Background:**

To investigate the skeletal development of HIV infected children through a morphological analysis of the cervical vertebrae (CV) in lateral cephalometric radiographs.

**Material and Methods:**

The sample consisted of 86 lateral cephalometric radiographs of male and female children aged between 6 and 14 years old. The radiographs were equally distributed in groups 1 (HIV infected children) and 2 (non-infected children, paired by sex and age). Two examiners analyzed the CV according to the method of Hassel and Farman (1995). Spearman correlation coefficient was used to associate age and skeletal development within groups, while Mann-Whitney test compared the skeletal development between groups.

**Results:**

The correlation of age and skeletal development in group 1 reached 0.17, 0.27 and 0.27 (*p* >0.05) for C2, C3 and C4, respectively, while in group 2 it reached 0.65, 0.54 and 0.60, respectively (*p* <0.001). Differences were not significant between groups (*p* >0.05).

**Conclusions:**

HIV infected and non-infected children showed a similar development of the CV. However, the weak correlation between age and CV development in HIV infected children highlights the need for careful decisions prior to therapeutic approaches – especially those founded on the prediction of skeletal development, such as maxillofacial surgeries, and orthopedic and orthodontic procedures.

** Key words:**Cervical vertebrae, growth and development, HIV, radiology.

## Introduction

Since the peak of the Human Immunodeficiency Virus (HIV) in 1997 (1) different strategies strived to promote health in face of this global threat. However, recent surveillance reports on the transmission of the HIV presented controversial rates worldwide (1). While in industrialized countries the number of HIV infected persons decreased, it remained stable or even increased among developing countries (2). In the last, pediatric HIV through vertical transmission (mother-to-child) figures as a social problem and an eminent cause of mortality (3). Over the last years, the Highly Active Antiretroviral Therapy (HAART) enabled longer life expectancy to children (4). On the other hand, the biological side effects of both HIV and HAART became daily challenges to be considered in order to provide better quality of life to children.

HIV infected children may manifest systemic alterations that impact on growth and development (3). In specific, the scientific literature has shown evidences that point towards a delayed growth (3-5). When associated with HIV, the delayed growth may manifest from endocrine and gastrointestinal disorders and recurrent infections (3). Differently, other authors (4) show that growth alterations may be related to aspects other than viral, such as poverty, tuberculosis and malnutrition (4). When associated with HAART, the eventual delayed growth may be an expression of the combination of toxic drugs (5). Protease inhibitors emerge in this context as substances known for their impact in osteoclast differentiation and bone metabolism (5). In this context, both the HIV itself and the HAART could play a role in the alleged delayed growth in HIV infected children.

In Dentistry, maxillofacial surgery, orthodontics and skeletal orthopedics figure as important fields in the treatment of functional and aesthetic growth disorders. The therapeutic approaches in these fields often require radiographic analysis is to screen skeletal morphology and to intercept skeletal disorders with optimal timing (6). The analysis of skeletal development through carpal radiographs was a common practice along the last decades (7). However, the additional exposure to radiation for the assessment of the hand and wrist consisted of an important limitation of the technique (8). Triggered by the increasing concern with radiation protection, the assessment of the cervical vertebrae (CV) through lateral cephalometric radiographs emerged as an alternative in the clinical practice (9,10).

Despite the advances in radiology for the assessment of skeletal development to therapeutic purposes, no study was carried to this moment investigate the radiographic development of the CV in HIV infected children. In order to support evidence-based practices and decisions in Dentistry, this study aimed to assess the development of the CV of HIV infected children ongoing HAART.

## Material and Methods

-Ethical criteria and protocol

The present study was conducted after the approval of the local Committee of Ethics in Research (protocol #1.479.700). All the ethical criteria for human research were followed, including the application of informed consent forms to the individuals sampled or their relatives.

-Study design and sampling

The sample consisted of 86 children divided in two study groups ( Table 1). Group 1 (n=43) was formed by male (n=18) and female (n=25) children aged between 6 and 14 years old (mean age: 10.26 ± 1.71 years). The children in this group were sampled by convenience after diagnosed with HIV by a Pediatrician via serological tests at the Department of Infectology at a pediatric hospital. All the children were vertically infected and received HAART since birth. According to medical records, their average TCD4+ lymphocyte count reached 35% and VL log10 of 3.25%, which represent controlled and ideal clinical conditions for asymptomatic children with HIV. Additionally to the diagnosis of HIV, the vertical infection and the use of HAART, the inclusion criteria for sampling group 1 also considered children that necessarily underwent treatment in both the Department of Infectology and the Dental Service at the University Hospital. The exclusion criteria consisted of children with clinical body malformations and previous medical history of fractures or surgeries in the neck. Group 2 (n=43) consisted of male (n=18) and female (n=25) children aged between 6 and 14 years old (mean age: 10.17 ± 1.68 years) not infected by the virus. This group was sample arbitrarily from the Department of Dentistry at the pediatric hospital to match group 1 by sex and age (e.g. for each HIV infected female aged 10 years old there was a non-infected female with the same age). Thus, the inclusion criteria consisted of sex and age matching group 1. The age matching process was performed allowing a maximum tolerance of 90 days between the children in both groups. The exclusion criteria in this group consisted of systemic diseases, body malformation and previous medical history of fractures and surgeries in the neck. In both groups the information on the exclusion criteria was extracted from the anamnesis, and from the medical and dental records.

**Table 1:**
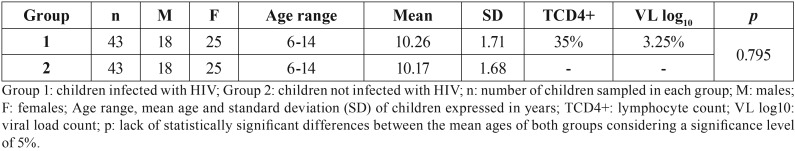
Demographic and clinical data of the children sampled in groups 1 and 2.

The children in both groups underwent treatment at the Dental Service at the University Hospital. Out of their dental records lateral cephalometric radiographs were retrospectively retrieved and used in the present study. All the radiographs were taken exclusively for therapeutic purposes. The radiographs were taken with an analog Veraview® (J. Morita®, Kyoto, Japan) panoramic device set at 62-66kV, 8-10mA and exposure time of 8-10 seconds. During 2.5 minutes, each radiograph underwent automatic processing (Revell®, São Paulo, SP, Brazil). Next, the radiographs were scanned in 300ppi using a HP Scanjet G4050® (Hewlett-Packard Co.®, Palo Alto, CA, USA) device and stored as .TIFF files.

-Data extraction

**Figure 1:**
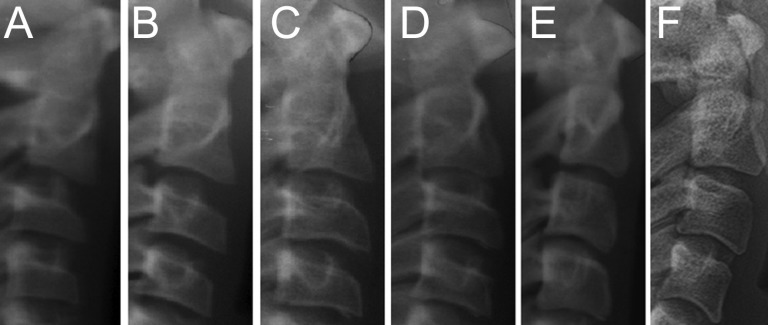
Radiographs of the six skeletal stages proposed by Hassel and Farman (9) for the development of the cervical vertebrae C2, C3 and C4. The images presented from A to F represent an approximation of the six developmental stages of the cervical vertebrae, namely the initiation (I), acceleration (II), transition (III), deceleration (IV), maturation (V) and completion (VI). According to the Hassel and Farman (9) in the initiation flat borders are observed in the inferior surface of all the vertebrae while the upper surface is tapered in the posteroanterior direction. In the acceleration the lower surface of C4 remains flat while in C2 and C3 it start becoming concave. The concavities in C2 and C3 become more distinct in the transition stage while in C4 it starts developing. Moreover, C3 and C4 present a rectangular shape. In deceleration all the vertebrae have a distinct concavity in the lower surface and the shape of C3 and C4 is closer to a square. In the maturation stage the concavities in all the vertebrae become more evident and C3 and C4 assume a squared shape. Finally, in completion, the concavities are evidently deep in the three vertebrae and C3 and C4 present vertical dimensions larger than horizontal dimensions. For orthodontic purposes, the authors (9) indicate that in stages I, II, III, IV, and V very significant, significant, moderate, small and insignificant growth may be expected, while in stage VI growth is completed (9). Images A-E were retrieved from the main sample, while image F was retrieved from the examiner agreement sample.

The digital radiographic files were provided to a trained examiner that proceeded with the analyses of the CV according to the method proposed by Hassel and Farman (1995) (9). The method is founded on a staging technique in which three CV, namely C2, C3 and C4, are classified into six developmental stages, namely I) initiation; II) acceleration; III) transition; IV) deceleration; V) maturation and VI) completion (Fig. 1) (9). The images were analyzed in a personal computer using software packages for image viewing and contrast manipulation (Adobe Photoshop CS5®, Adobe Systems®, San Jose, CA, USA). The examiner was blind for the sex, age and health condition of the patient in each radiograph. In order to assess the examiner reproducibility, intra- and inter-examiner agreement tests were performed. In the first, the main examiner analyzed 25 lateral cephalometric radiographs following the method previously described and performed a second and a third analysis 7 (n=25) and 14 (n=25) days later, respectively. In the second, an additional examiner was included to analyze the same 25 radiographs. All the radiographs used in the examiner agreement tests were did not belong to the main sample. They were collected retrospectively from the university database.

-Data analysis

T-test for independent samples was used to compare the patients’ age in groups 1 and 2. This procedure was necessary to validate the sample pairing process on matching the patients by their age. Spearman correlation coefficient was used to associate the age of patients within each group with the respective developmental stage of their CV. Mann-Whitney test was applied to compare the developmental stages of the CV between groups with and without sex stratification. Intra- and inter-examiner agreement tests were quantified with Weighted Kappa test. Statistical tests were performed with Statistica 13.0 (StatSoft Inc., Tulsa, OK, USA) software package and their statistical significance was set in 5%.

## Results

The t-test for independent samples pointed no differences statistically significant between the age of the patients sampled in group 1 and 2 (*p*=0.795). More specifically, the mean age among HIV infected children was 10.26 years (±1.71) while in non-infected children the mean age was 10.17 years (±1.68). When the assessment of sample matching was performed comparing the age of males and females in each group the lack of differences statistically significant remained (group 1: *p*=0.192; group 2: *p*=197).

Spearman correlation coefficient showed a positive correlation (r>0) between the age and the developmental stage of the CV within groups 1 and 2. The coefficient of determination in children infected with HIV ranged from 0.17 to 0.29, while in children not infected with the virus it ranged between 0.54 and 0.65. The correlation was statistically significant for all the CV in the age of group 2 (*p*<0.001), while in group 1 it was not (*p*>0.05) ( Table 2).

**Table 2:**
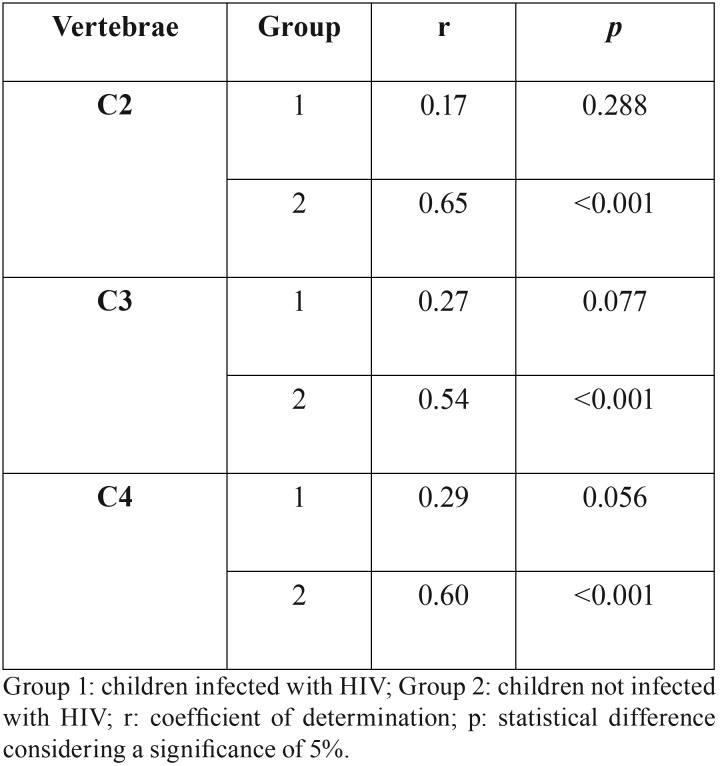
Correlation between the developmental stages of the cervical vertebrae and the age of patients sampled within group 1 and 2.

Mann-Whitney test showed no difference statistically significant between children infected and not infected with HIV for all the CV ( Table 3) (*p*>0.05). The most similar distribution of the developmental stages between groups was observed for C2 (*p*=0.810), followed by C4 (*p*=0.530) and C3 (*p*=0.513).

**Table 3:**
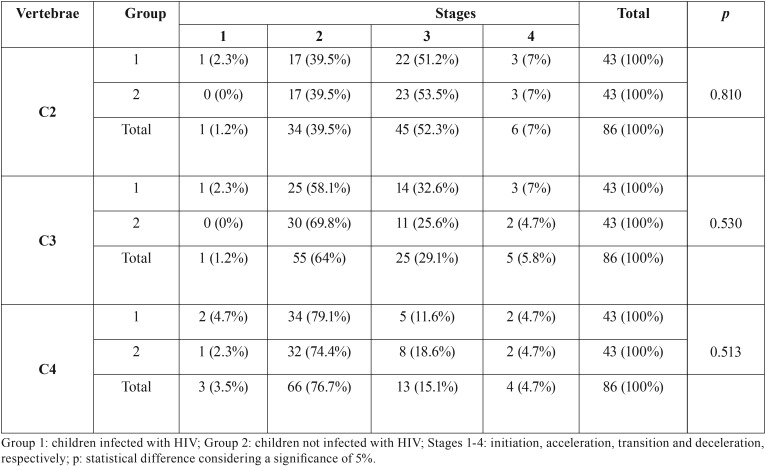
Distribution of the developmental stages of the cervical vertebrae C2, C3 and C4 in children infected and not infected with HIV and the respective comparison between groups.

Comparisons on the distribution of developmental stages between males and females did not reach statistically significant differences (*p*>0.05). In children infected with HIV the most similar distribution of the developmental stages was observed in C4 (*p*=0.913), followed by C2 (*p*=0.798) and C3 (*p*=0.335). In children not infected with the virus the similarity of distribution followed the same pattern (C4: *p*=0.990; C2: *p*=0.652; C3: *p*=0.400) ( Table 4).

**Table 4:**
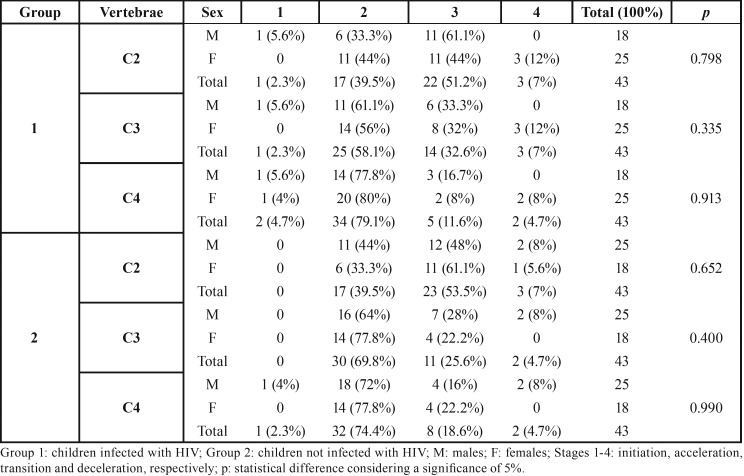
Distribution of the developmental stages of the cervical vertebrae C2, C3 and C4 in male and female children infected and not infected with HIV and the respective comparison.

Intra- (0.79) and inter-examiner (0.85) agreement tests reached great and excellent outcomes, respectively.

## Discussion

Equity for accessing proper health treatment is a global concern, especially regarding maxillofacial orthopedics and orthodontics (11-16). In this context, HIV infected children figure as a population under the need of major attention on health care. Screening the skeletal development in this population is an important step to guide surgical and orthopedic procedures. Based on it, the present study was designed to investigate the CV development in lateral cephalometric radiographs of HIV infected children.

In order to design a systematic and standardized radiographic assessment of the development of the CV, sample matching was performed. Similar strategy was previously performed in studies on craniofacial (17) and dental (18) development of HIV infected children. To accomplish this task a reference sample (group 2: non-infected children, mean age: 10.17 years ±1.68) with age and sex compatible with the study group (group 1: HIV infected children, 10.26 years ±1.71) was selected. Success in sample matching was confirmed with the lack of statistically significant differences between the mean ages of both groups. This procedure contributed to avoid age-related bias in the comparison between groups.

The patients’ age (independent variable) was tested based on their correlation with the developmental stages of the CV (dependent variable) in both groups. Positive correlations were observed ( Table 2), but they were statistically significant (*p*=0.001) only in group 2. The coefficient of determination (r) expressed in group 2 ranged between moderate and strong (from 0.54 to 0.65), which indicates that in group 2 a high proportion of variance in the developmental stages is detectable from the chronological age. Similar outcomes were observed in previous studies correlating the method of Hassel and Farman (9) (1995) with the chronological age in populations with no systemic diseases (18,19). In HIV infected children, growth may be affected (20) leading to a delayed skeletal development (21). Consequently, the correlation between the developmental stages of the CV and age may be altered. Very weak and weak coefficients of determination in group 1 (from 0.17 to 0.29, *p*>0.05) confirm this statement. Considering the aim of this study, these outcomes point towards the need for careful steps into procedures that involve the prediction of growth from CV in HIV infected children. The weak correlation between age and the stages of skeletal development in group 1 indicates that in the clinical practice the radiographic assessment of the cervical vertebrae may be unreliable to predict maxillofacial growth and consequently planning therapeutic approaches.

Despite the lack of statistically significant correlation between the stages of CV development and the age of children infected with HIV (group 1), the skeletal development between groups did not differ significantly (*p*>0.05) ( Table 3). Additionally, comparisons based on sex also did not reach statistically significant differences (*p*>0.05) ( Table 4). To our knowledge, no study was previously designed to assess the development of the CV in HIV patients. The available scientific literature is scarce and focuses on the radiographic assessment of the hand and wrist (21,22), the teeth (19,22,23) and the craniofacial bones (18). In the clinical practice, information on the skeletal development of HIV infected children through the CV is relevant for any therapeutic approach that requires that analysis and the prediction of growth. More solid and palpable application is found in dentomaxillofacial orthodontics and orthopedics, in which lateral cephalometric radiographs became part of the treatment planning.

The exact reason why the skeletal development is affected in children with HIV is complex and uncertain among authors in the scientific literature. In one hand, studies show that HIV may cause hypovitaminosis D (24). Vitamin D has a role in maintaining Calcium available in serum levels (25) and a consequent part in avoiding risk of bone alterations. Long-term lack of balance of vitamin D and Calcium could trigger alterations in the development of the CV in HIV patients. However, in the present study, all the patients sampled were children controlled with HAART. Their clinical condition with drug control was adequate based on TCD4+ and VL log10 count and for this reason evident bone alterations in lateral cephalometric radiographs was not expected (based on solely on HIV itself). In the other hand, the HAART combines several drugs that are toxic and may have association with disorders in bone metabolism (26). These drugs are known for their impact in osteoclastic activity and potential association with osteoporosis and osteopenia. Among these drugs, protease inhibitors figure as potentially influent in decreasing bone mineral density (26). Additionally, these drugs also may have a potential impact over the availability of vitamin D and Calcium. Specifically, *in vitro* studies show a suppression of 25- and 1-alpha-hydroxilase – important components on the synthesis of vitamin D (27,28). Again, the lack of vitamin D in optimal levels has a negative impact in Calcium homeostasis. Radiographic bone alterations could be expected in the patient sampled is this study because all of them were infected by HIV and were controlled with HAART since birth. However, tracking the potential influence of HAART in HIV infected children and adolescents or even screening their laboratory blood tests for vitamin D count is a task for further investigations.

Other investigations are also encouraged to overcome the limitations found in the present study, especially the small sample in the control group. Control groups have an important part in providing more reliability to the research methods (29). Matching case and control groups in a ratio above 1:2 (preferably near to 1:4) would benefit future studies with a major statistical power (30). Following the sampling approach of the present study, case and control matching by sex and age should be maintained to avoid eventual and inherent bias. Additional alternatives for scientific investigations may include homogeneous sampling in different age limits and the use of other radiographic methods for the assessment of skeletal development. These methodological improvements would enable not only the assessment of skeletal development but also age estimation between HIV infected and not infected children.
